# Audience-Dependent Explanations for AI-Based Risk Management Tools: A Survey

**DOI:** 10.3389/frai.2021.794996

**Published:** 2021-12-21

**Authors:** Branka Hadji Misheva, David Jaggi, Jan-Alexander Posth, Thomas Gramespacher, Joerg Osterrieder

**Affiliations:** ^1^ ZHAW, School of Engineering, Institute of Data Analysis and Process Design, Winterthur, Switzerland; ^2^ ZHAW, School of Management and Law, Department Banking and Finance, Winterthur, Switzerland

**Keywords:** explainable AI, responsible AI, artificial intelligence, machine learning, finance, risk management

## Abstract

Artificial Intelligence (AI) is one of the most sought-after innovations in the financial industry. However, with its growing popularity, there also is the call for AI-based models to be understandable and transparent. However, understandably explaining the inner mechanism of the algorithms and their interpretation is entirely audience-dependent. The established literature fails to match the increasing number of explainable AI (XAI) methods with the different stakeholders’ explainability needs. This study addresses this gap by exploring how various stakeholders within the Swiss financial industry view explainability in their respective contexts. Based on a series of interviews with practitioners within the financial industry, we provide an in-depth review and discussion of their view on the potential and limitation of current XAI techniques needed to address the different requirements for explanations.

## 1. Introduction

AI has developed into a wide-ranging tool that allows us to fundamentally rethink how data is integrated, analyzed, and used for decision-making. Every day, we experience it when we scroll through our Twitter Feeds, get movie suggestions on Netflix, or discover new products on Amazon. With the increase in computing power and the advances in computer science, the range of possible models to implement expanded significantly from simple linear models to highly complex methods. The latter can deal with the ever-growing dimensionality of the input space and thus provide a good basis for decision-making (e.g., Deep Neural Networks (LeCun et al., 2015)). Businesses are increasingly turning to AI solutions as emerging toolsets promise to deliver faster and more accurate results compared to humans.

**FIGURE 1:**
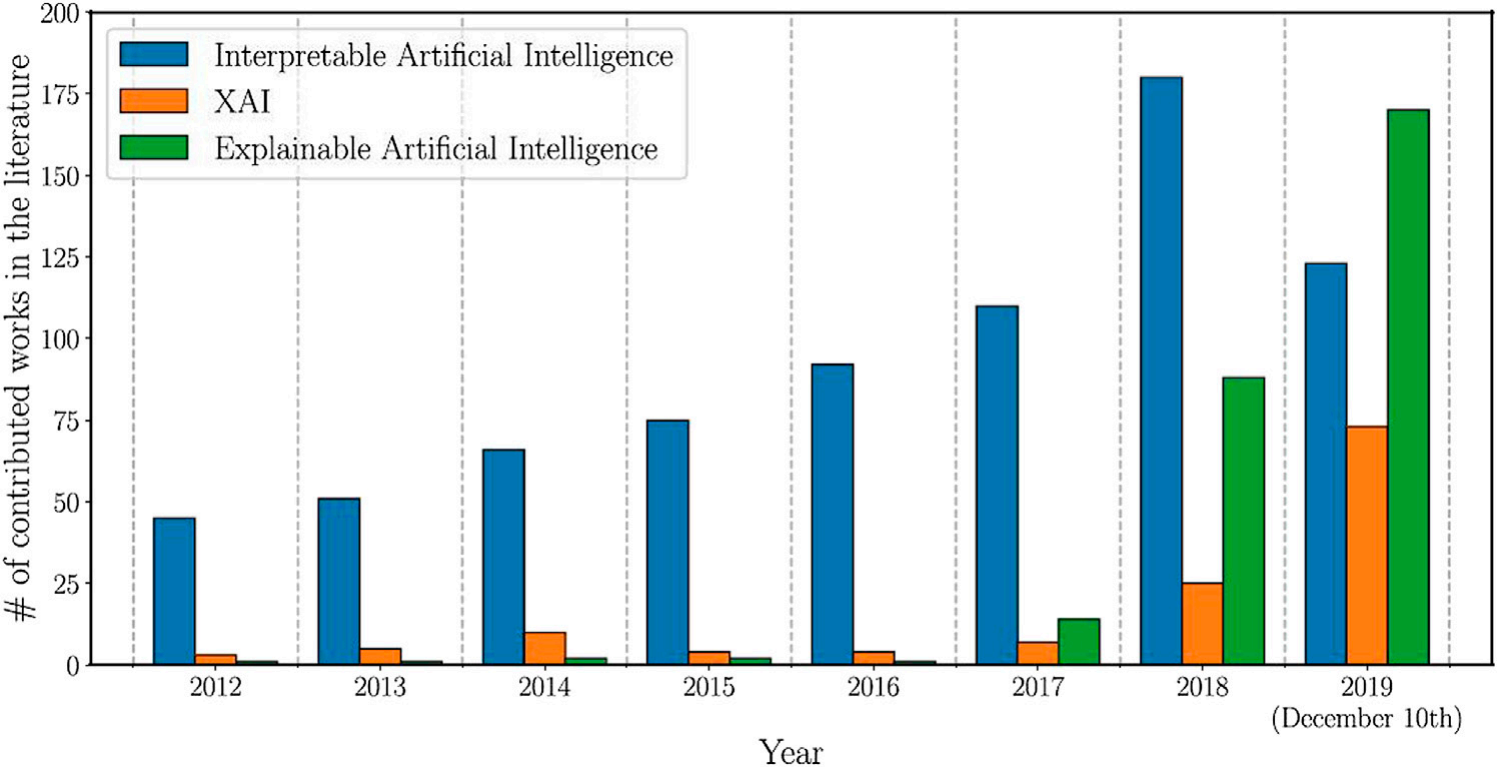
Number of total publications related to the terms “Interpretable Artificial Intelligence”, “XAI”, and “Explainable Artificial Intelligence” between 2012 and 2019 (Arrieta et al., 2019).

These benefits offered by AI-based systems became even more relevant because of the COVID-19 pandemic. (Costello and Rimol, 2020) reveals that 66% of organisations have either increased or held their investments in AI since the beginning of COVID-19. Figure 1 displays the rising attention of the academic sector to the fields of Explainable AI over the last years.

In addition to the higher complexity, the speed of development increased exponentially as well. Business considerations mainly drove this development as leading companies need to stay on top of new technological developments. The acceleration in AI development is necessary to remain competitive among other institutions and for promotional purposes. The new tools promise to provide insights into customer behaviour, spending trends and provide the knowledge to customise products and price risk. Nowadays, some models need almost no human intervention to fine-tune (Gijsbers et al., 2019).

**AI in Finance.** Following a detailed report by the (Financial Stability Board 2017), AI and machine learning (ML)-based solutions are being adopted for a variety of purposes across the financial industry, ranging from sentiment indicators and trading signals to anti-money laundering and fraud detection. A review of the literature concerning the application of ML in the financial sector reveals three main research areas: 1) portfolio optimization, 2) risk management, and 3) compliance.

1) In the context of portfolio optimization, the potential of sophisticated methods has been the subject of discussion in the literature for the past 2 decades (Villhauer 2021), starting with the work of (Streichert 2001), who investigated practical issues with evolutionary algorithms, such as parameter optimization and constrained portfolio selection. More recently, the literature is counting an increasing number of works that propose ML and deep learning (DL) models for the task of automated stock trading. One pioneer work in this context is that offered by (Kimoto et al., 1990). The authors proposed a modular neural network to predict stock trends. Since (Kimoto et al., 1990)’s work, many other researchers have proposed neural network architectures for portfolio optimization tasks (Zhang et al., 2020). Recently, the use of reinforcement learning in stock market prediction has shown great promise as demonstrated empirically by (Bertoluzzo and Corazza 2012) and (Huang 2018).
2) Turning to the second area of application, credit risk evaluation is a very challenging and important task in financial analysis (Wu, Gao, and Xu 2021). Many methods, including ML and DL-based approaches, have been applied to tackle this task. As early as in the 1990s, researchers proposed ML-based techniques for determining the creditworthiness of applicants (Henley and Hand 1996), (Piramuthu 1999) and (Lessmann et al., 2015). More recent studies on the same topic propose classification techniques that can significantly improve the model’s accuracy, ranging from a single classifier method (decision trees, support vector machines (SVMs), neural networks (NNs), etc. (Turiel and Aste 2020), (Pławiak et al., 2020)) to an ensemble method (Florez Lopez and Ramon Jeroni, 2015).
3) Finally, efficient financial regulation is paramount to the future success of the financial sector. A study by (Batkins et al, 2016) found that the Dodd-Frank Wall Street Reform led to $36 billion in regulatory cost and 73 million paperwork hours since it passed in 2010. Consequently, a key area of research is how to employ innovative technologies to facilitate regulatory compliance for financial service providers. As stated by (Ju et al., 2017) ML creates great possibilities when applied to compliance. Namely, ML is suited for analyzing large sets of unstructured data, which in turn means it can help improve the interpretation of low-quality data outputs from payments systems. Furthermore, ML-based solutions can lead to more accurate predictive models needed for stress testing (Petropoulos et al., 2020).

**Challenges.** Despite all the enthusiasm, the real-world implementation of AI remains challenging. Namely, AI solutions are often referred to as “black boxes” because, typically, it is difficult to trace the steps the algorithm took to arrive at its decision. This unclarity is mostly because AI models are built on complex logic, often with thousands of parameters interlinked with nonlinear dependencies. This property is considered one of the biggest challenges in implementing AI solutions in practice. It makes the decision-making process intransparent and often incomprehensive even to the developers of the tools.

To emphasize the relevance of this challenge, we discuss an interesting thought experiment that was posed before the announcement of the winners of the 2018 NeurIPS Explainable Machine Learning Challenge. The participants were asked to think about the following situation (Rudin and Radin 2019).

*‘Suppose you have a tumor and need surgery. Would you rather trust an AI surgeon who cannot tell anything about its inner workings but has a 2% chance of making a fatal mistake or a human surgeon who can explain every step in detail but has a 15% chance of making a fatal mistake?’*

The AI’s 2% chance of fatality is the superior of the two choices. Nevertheless, even with the better choice in terms of risk, we would still feel uncomfortable as we need explanations to trust the decision. Hence, in this case, there emerges a need to understand the machine’s inner workings, which ultimately leads to the decision. This example shows one of the significant drawbacks of today’s complex AI architectures and showcases the trade-off between efficiency and explainability we currently need to make. Furthermore, it also shows the human need for ever-higher-quality decision making versus the desire to understand and trust.

The challenge of explainability is particularly relevant for Swiss financial intermediaries as they are subjected to the General Data Protection Regulation (GDPR). This regulation took effect in 2018 and places restrictions on automated individual decision-making. It provides a ***right to an explanation***, enabling users to ask for an explanation as to the automated decision-making processes affecting them.

**XAI in Finance.** As a result of such rising concerns, the concept of XAI emerged, introducing a suite of techniques attempting to explain to users how the model arrived at a particular decision (i.e., (Lundberg et al., 2019), (Arya et al., 2019), (Molnar et al., 2020), and (Sokol and Flach 2020)). In terms of the taxonomy of XAI methods, the literature offers many papers that provide a comprehensive overview of the existing research in the field of XAI (see, e.g., Arrieta et al., 2019).

XAI offers the potential of providing insights into model behaviour through various concepts such as feature importance scores or counterfactual explanations (Bhatt et al., 2020). In general, methods are considered in view of two main criteria (Linardatos et al. 2021): 1) the type of algorithm on which they can be applied (model-specific vs. model-agnostic) and 2) the unit being explained (if the method provides an explanation which is instance-specific then this is a local explainability technique and if the method attempt to explain the behavior of the entire model, then this is a global explainability technique). For the most part, these techniques, introduced through academic and industry efforts, aim to address the need for explainability of AI developers and engineers. However, the value chain in the financial industry includes many stakeholders, all of which might have different explainability needs.

Moreover, the literature outlines other challenges associated with XAI starting with the inconsistency of the term “*explainable*” among different sources. The lack of standards comes down to XAI being a relatively new field of research. Hence there is no established standard terminology. Similar problems arise when comparing different XAI methods. Currently, no standard metrics exist to determine the notion of explainability as it is highly task-specific. (Arrieta et al., 2019) argue the need for comparable XAI methodologies in order to identify methods that are high performing.

These issues point to the slight decoupling between what is being developed in terms of explainable systems and the actual needs of the industry.

**Purpose of study.** This study explores how different stakeholders within the Swiss finance/financial industry view the potential of ML and the need for explainability. By explicitly identifying the different needs of explainability of the key stakeholders within the Swiss financial sector, we aim to close the gap that exists between explainability in theory and in practice. This we do by mapping out the requirements of explainable systems that can improve the trustworthiness of automated decision-making. The paper is structured as follows. In section 2, we describe the research design employed in the paper. Section 3 presents the results from the conducted interviews, and section 4 summarizes the discussion and provides an outlook. It is important to note that in this work, we use the terms AI and ML interchangeably.

## 2 Research Design

### 2.1 Structure of the Interviewee Audience and the Interviews

Following the example of (Holstein et al., 2019) and (Bhatt et al., 2020), we study how the industry looks at the recent developments in ML and the need for deploying explainable AI solutions. The interviewees were representatives from several FinTech companies, one bank, one large association and one large insurance company, which all operate in Switzerland. The interviewees were selected to represent the various stakeholders within the financial sector and hence were composed of ML engineers, risk and legal experts and higher management. A summary of the interviewees’ composition is presented in Table 1. Some institutions asked to stay anonymous. Therefore, they are not referred to in any way in the text nor included in the acknowledgements. With each representative institution, we held an hour-long interview. The responses are consecutively summarized as the viewpoints of two broad classes of stakeholders, i.e. those with and without a technical role in building and deploying AI (and XAI) systems. A total of seven semi-structured interviews were conducted.

**TABLE 1:** Composition of the interviewees participating in the seven semi-structured interviews.

Category	# Participating institutions	Location of participating institutions	# Interviewees	Background/ Role of interviewees
FinTechs	4	Zug (1), Geneva (1), Zurich (2)	3 × 1, 1 × 2	ML engineering, management
Banks	1	nationwide	2	Management, legal experts
Insurance companies	1	nationwide	2	ML engineering, risk experts
Banking associations	1	nationwide	2	Management, legal experts
**Total**	7		11	

**Source: Own Data.** With each representative institution, we held an hour-long interview. The responses are consecutively summarized as the viewpoints of two broad classes of stakeholders, i.e. those with and without a technical role in building and deploying AI (and XAI) systems. A total of seven semi-structured interviews were conducted.

### 2.2 Discussion Points and Objectives of the Interviews

The interviews were divided into three sections covering different aspects of the use of AI in industry. Specifically, the discussion points were:

• **The potential benefits presented by machine learning (ML) in finance;** In the first section, we discussed the current use of ML within the finance sector. The main objective in this context is to understand the state of ML in finance and the primary motivations for its adoption.
• **The main barriers for wider adoption of ML-based solutions in finance;** In the second section, we focus on understanding the main hurdles companies face when deploying ML and the relevance of explainable and interpretable solutions.
• **Deployment of XAI methods: state of use and explainability needs;** The final section focuses on understanding the requirements of explainable systems that will improve the trustworthiness associated with automated decision-making.

## 3 Results and Analysis

### 3.1 Machine Learning in Finance

*“A century ago, factories electrified without rethinking their production lines and therefore saw no productivity benefits. In much the same way, machine learning technology without management and organizational change will be ineffective.”*

- *Erik Brynjolfsson, Professor at MIT Sloan School of Management (Johnson 2019)*

Given the growing volumes of data, increases in computational power and the continuous advancement of methods, ML is gaining significant momentum in the financial industry. The interviewees with a more technical role within their respective institutions argued that ML introduced significant changes in the modeling paradigm. This change enabled model developers to switch from simple mathematical approaches, which are valid only in structured, well-defined data sets, to methods based on learning algorithms. The methods based on learning algorithms can be applied to complex, unstructured data. As a result, ML experts are often in favor of the wider adoption of ML-based solutions to the various processes in financial intermediation. The solutions have applications ranging from fraud detection and customer analysis to modeling risk and optimizing market-making and hedging strategies. Representatives from the management-level positions also argued in favor of the advantages that sophisticated algorithms can bring to the overall performance of financial service providers. The benefits are both in terms of cost savings and increased revenues.

Nevertheless, in their viewpoint, the difficulty lies in the vast range of models available for implementation. The broad range of models makes the cost-benefit analysis of individual solutions challenging to assess. Furthermore, the business executives pointed to various other open questions concerning AI’s application in finance. These open questions concern the future profitability of investments in AI research and development. Another question addresses the resource intensity of implementing an effective AI strategy. Executives are also unsure about organizational changes that are necessary to improve the performance of existing processes.

In terms of the specific applications of ML in finance, the interviewees identify three distinct areas: 1) risk management, 2) algorithmic trading, 3) fraud detection and compliance. Interviewees representing the fintech credit sector identified risk management as one of the key areas where ML can lead to significant benefits. The developments in the computers’ processing capabilities have enabled model developers to build credit scoring models using methods such as Deep Learning, Random Forest and Gradient-Boosting Machines, and ensembling techniques that combine the outputs of multiple models. These methods can, in turn, be applied to problem sets in different areas of credit risk management, including risk scoring and monitoring, provisions, regulatory capital allocation and others. In addition to credit risk management, interviewees discussed the use of ML systems for improvements in systematic trading. Specifically, interviewees suggest that ML allows fast and automated trading decisions at the best possible prices. Finally, a key area identified by interviewees where ML can make an impact is fraud detection. In essence, fraud is an adaptive crime. Hence, there is a strong need for dynamic algorithms which can learn from previous data. Within the ML domains, the interviewees with a technical role indicated that algorithms for anomaly detection had been proven very successful in identifying unusual patterns in large datasets.

### 3.2 Barriers for Wider Adoption of AI

In the second section of the interviews, we capture the main hurdles companies face in deploying ML and the relevance of explainable and transparent solutions. The interviews revealed numerous challenges affecting the wide-scale adoption of ML systems in finance. These are discussed below, separated into internal factors (conditions that can be affected and changed within the organization) and external factors (constraints imposed on the organization by the environment).

#### 3.2.1 Internal Factors

**Management’s understanding of the value generated by AI.** A subject most often voiced by the interviewees from the business side concerning the barriers for wider adoption of AI-based solutions is the unclear value proposition of AI. This issue was particularly relevant among the interviewees from incumbent firms who stated that AI-driven use cases often come with an uncertain return on investment, which is a key point of interest for senior management. Interviewees also noted that legacy IT infrastructure used by established financial service providers lack the necessary flexibility and capacity to support the various computing requirements associated with running state-of-art ML algorithms and produce output in real-time.

**Cultural factors.** Another barrier to AI implementations in finance is associated with organizational culture. Representatives from the business side argued that weak corporate culture, i.e. ineffective communication and coordination, political factors, limited use of best practices, and lack of management support, often represent crucial barriers.

#### 3.2.2. External Factors

**Data access and quality.** The interviewees representing the technical role within finance service providers identified data quality as one key obstacle to AI’s implementations. ML models learn iteratively, which requires model developers to have access to large sets of high-quality data to ensure a proper problem representation. Data quality issues themselves can take several forms:

• **Data sparsity:** Not observing a sufficient quantity of data, especially when dealing with low frequencies of occurrence and/or high-dimensional parameter spaces. This problem is, for example, highly relevant in the domain of FinTech credit, where marketplace lenders and credit refinancers are unable to observe sufficient information on the platforms’ participants to build and calibrate accurate ML-based models.
• **Data variety:** Collecting data from multiple data sources and in different formats requires significant effort in terms of data integration which remains one of the biggest challenges in computer science.
• **Missing data and noise:** Missing information is a factor that degrades model performance. Therefore, efficiently handling missing information by model developers becomes a crucial step toward the broader application of AI solutions. Moreover, interviewees also pointed out the adverse effects of random perturbation in the data. Depending on the extent to which it is present in the data, noise can result in several problems ranging from slow training to inaccurate predictions.
• **Bias in data:** Big data is, by definition, highly heterogeneous and generated by different entities, operating under varied conditions and environments. These differences can often lead to biases in the data. Models trained on such information may lead to unfair and inaccurate predictions.

**Access to knowledge.** Representatives of the management-level positions argued that a relevant hurdle for the wide-scale adoption of automated, AI-based solutions in finance is the lack of qualified talent. AI implementations in finance require specialist skills across various disciplines, including computer science, domain knowledge and advanced methods. This finding stands in line with conclusions reached by two other studies ((Bschor and Budworth 2018) and (Ryll et al., 2020)). The challenge is further increased as for financial service providers, the competitive landscape of the future will include many entities and geographies. Another challenge are “Big Tech” firms that have persistently engaged in “talent grabbing,” i.e., attracting top researchers straight out of academia with high salaries.

The technically focused interviewees suggested that teams that focus on model development and implementation are typically relatively small and work independently from the day-to-day operations. They work in an experimental mode, which further limits the ability of developed solutions that fundamentally change how the business works.

**Fast evolving field: methods and regulation.** A common thread among the interviewees when discussing barriers for wider adoption of AI systems in finance is the overall dynamic nature of the field. On the modeling side, developers often struggle to stay up to date with all the novel ML methods introduced in the literature at an increasing rate. This not only impacts the models being developed but also the communication with the executive side. Furthermore, the interviewees with a technical role also pointed out the lack of cross-language and framework support. Namely, since ML models are fast-changing, a wide range of programming languages and tools are used. A FinTech representative mentioned that frequently a pipeline would start with Python and then use different capabilities available in R or other languages. This lack of consistency, in turn, means that projects very quickly become difficult to track and scale. On the business side, interviewees also discussed the instability in terms of the regulatory framework around novel technologies. Namely, the financial industry operates under a comprehensive regulatory framework which until now followed an evident sense of who is acting, with what intentions and where the action takes place. With AI-based tools being part of the decision-making processes at financial service providers, various legal questions emerge: What recourse should be available for individuals who have been denied insurance or a loan based on an algorithm’s decision? Who is responsible for a trading loss resulting from a sophisticated algorithm incorporating market data and news coverage? How do regulators remain technology-neutral and still ensure customer protection?

**Explainability**. All seven interviewees identified explainability as one highly relevant barrier for the wider adoption of AI in the financial sector. As discussed previously, black-box models are created directly from data by an algorithm. Hence often, it can be very challenging to trace the steps the algorithm took to arrive at a particular decision. Representatives from the business side pointed out that this challenge is particularly relevant given the General Data Protection Regulation (GDPR). Model developers also stressed the need for explainable AI systems suggesting that one cannot trust accuracy alone. In practice, it is essential to verify that the high model accuracy results from proper problem representation for a given task: The model needs to capture the true dependencies rather than exploiting noise in the data.

The interviewees further stated that this explainability problem becomes particularly relevant given the developments associated with big data. Namely, big data creates more dimensions, resulting in more interactions between variables and higher complexity that humans cannot easily understand. In this context, interviewees with a more technical role suggested having solutions outside of post-hoc explainability. Namely, for specific models, one can use feature importance plots to provide information on how important a specific feature is for the prediction. For example, feature importance is calculated for a random forest classifier as the decrease in node impurity is weighted by the probability of reaching that node. In practical terms, the more critical a feature is, the higher in the tree on average one will find it.

Nonetheless, these solutions still lack full transparency regarding the relationship between the input space and the outcome. Specifically, feature importance plots fail to identify the direction of the relationship, which is crucial for interpretability hence the need for post-hoc XAI methods. The literature distinguishes between two main approaches for post-hoc explainability. First, those designed for their application to ML models of any kind, and second, those designed for a specific ML model and thus cannot be directly extrapolated to any other ML learner (Arrieta et al., 2019). Among the emerging techniques, two frameworks are widely recognized as the state-of-the-art in machine learning explainability. Those are: 1) the LIME framework, introduced by (Ribeiro et al., 2016) and 2) SHAP values, introduced by (Lundberg and Lee, 2017). Both methods shed light on the inner workings of black-box models, thereby explaining the reasoning behind the predictions (Misheva et al., 2021). LIME is a local surrogate model based on a weighted linear regression that explains the features that drive the prediction for a specific observation. Yet another possible approach for explaining predictions comes from cooperative game theory. Shapley values are a method for assigning payouts to players depending on their contribution to the total payout. Since ML models usually have a high-dimensional input space, the computation of Shapley values for each feature instance can be very computationally intensive. To address this challenge, (Lundberg and Lee, 2017) introduce KernelSHAP, a method based on the weighted linear models, which allows the calculation of Shapley values with much fewer coalition samples. The key difference between the two approaches is related to weighting the instances in the regression model. Namely, LIME uses weights that correspond to the actual proximity to the original instance. In contrast, SHAP weights the samples based on the coalition’s weight in the Shapley estimation.

In the following subsections, we discuss in detail the viewpoint of practitioners concerning the utility of such state-of-the-art XAI frameworks in the context of finance problem sets, specifically looking at two main aspects: 1) human-centric issues; and 2) technical/mathematical issues.

*Human-centric issues.* On what is considered a suitable explanation, all seven interviewees stressed the strong audience-dependency of the problem. Namely, the form of the explanation will significantly depend on the information that needs to be provided and the capacity of the receiver to interpret it. In this context, we identify two broad groups of stakeholders within the XAI domain:

• Model Developers
• Non-Technical Audience

The non-technical audience includes all entities with no specific understanding of the methodology but occupying a role within the finance value chain that either validates or approves the AI solution (business executives, legal and risk audit teams, regulators) or is directly affected by the outcome (end users).

This categorization is somewhat consistent with those identified in similar studies in the literature (i.e., (Berg and Kuiper, 2020) and (Bhatt et al., 2020))

The interviewees further stressed that different stakeholders belonging to these two broad groups would have different needs in terms of explainability. Specifically, model developers are interested primarily in the performance and stability of predictions. Key objectives of the explainability function in this context would be model debugging, robustness and accuracy. Business executives who have to approve the deployment of models that directly affect end users must ensure that the system is transparent and in line with existing regulations. In this context, the prime focus is to develop models that provide explanations based on robust justifiable factors: Executives are interested in the overall behavior of the model and whether it is in line with financial logic. End-users also belong to the non-technical audience who are directly affected by the outcome of the AI model. The interviewees suggest that these stakeholders are primarily interested in why the model has arrived at a certain decision for their unique case and what can they do in the future to obtain a different outcome.

All seven interviewees suggested a clear need for XAI methods to deliver the explanation needed by stakeholders occupying a specific role. Put differently, XAI techniques have to integrate human-centric factors in the development phase. Currently, the research community of XAI has largely focused on developing methods that ‘reverse engineer’ the decisions of complex machine learning models, extracting relevant input features and their corresponding contribution to the predictions. These methods can provide valuable inputs for model developers as they enable the extraction of valuable information concerning the model’s overall logic and dependence on relevant features. However, their utility does not extend to all relevant stakeholders. Interviewees suggested that state-of-art explainability techniques (like LIME, SHAP etc.,) are predominantly employed by model developers as a robustness indicator rather than providing information to end-users or other non-technical stakeholders. This finding is in line with the results by (Bhatt et al., 2020), who find that ML engineers and data scientists use local explainability techniques to audit models before deployment.

*Technical/mathematical issues.* Moving on from the human-centric issues to the deployment issues, the interviewees familiar with the deployment of XAI methods suggested that existing implementations might not be best suited for the practical constraints in which financial institutions operate. One interviewee indicated that most state-of-art XAI methods require one point of data access which can be very challenging in real settings where data access is restricted to specific teams within the organizational structure. Furthermore, as confirmed by a recent paper by (Kumar et al., 2020), problems arise from the estimation procedures used for specific post-hoc explainability techniques. In the context of the SHAP estimations, both marginal and conditional value functions have their distinct challenges. If features are correlated, sampling from the marginal distribution to simulate that a certain feature is missing from a given coalition may lead to meaningless feature values for certain instances. This challenge is prevalent in all permutation-based interpretation methods (Aas et al. 2021). On the other hand, the conditional value function induces different challenges, including computational complexity. For example, the exact computation of SHAP values requires a significant number of approximations for high dimensional input space (Kumar et al., 2020). Other challenges are feature selection issues. An example of this challenge is that features must be selected carefully, as redundant features might get a non-zero SHAP estimate if they are correlated with another feature that influences the prediction.

## 4 Discussion and Outlook

This survey aims to answer a simple yet highly relevant question: With the progress made in AI and big data statistics and with the potential and all the benefits the adoption of AI-based solutions promise, why do we not see a wider, if not massive, utilization and integration of AI in risk management in particular and in finance in general?

The interviewees stated that this lack of widespread adoption of AI systems comes, in part, as a result of various internal and external factors ranging from legacy IT landscape, which is not accommodating for advanced analytics, to data quality issues and limited access to suitable talent.

Next to the more detailed findings discussed in Section 3, one aspect has been quite prevalent in the interviewees’ feedbacks we collected and analyzed: the element of trust. When it comes to accepting to trusting a decision, humans need to understand how this decision came about. This fact becomes even more relevant in all matters related to finance: the very translation of the Latin origin of the word “credit,” credere, literally means “to trust” or “to believe.” In any business relationship, this trust is established over time, based on a shared and common basis of culture, understanding, mindset, and interest. If I can understand why you decide or act this way, and if I observe your behavior consistently over time, I start trusting you. But culture, understanding, and interest never can be shared with any machine—at least not yet. For simple statistical machines, like linear regression, at least the mindset, the “how they (have to) think,” is predefined—but even in this simple example, humans do not trust the results if they contradict common expectation, rational, or intuition. For more advanced statistical machines operating in a nonlinear way on a high-dimensional parameter space, even “sharing the mindset, the way how to think” is no longer possible—so why then trust this black-box AI?

Our interviewees agree that thus two aspects are of utmost importance for accepting AI-based decision-making processes: First, decisions or results of the process need to be consistent over many decision instances - over time and cross-sectionally spanning the possible parameter space. Second, a rationale needs to be inferable based on the process, or at least from the results of the process, that can be understood and that “makes sense” in the domain of application—in other words, the decision, the result needs to be explainable in a relevant context. This central insight holds no matter whether the target audience is a data scientist, a business engineer, a user, a manager, a customer, or a regulator: they all need to be able to have faith, to trust in the reliability and the correctness of the AI engine in question - albeit on entirely different levels of complexity, as outlined in Section 3.

Consequently, a software tool addressing the need for explainable AI needs to cater for these different levels of complexity as well as for the different mindsets the targeted audiences will have: As we have learned from the feedbacks of this survey’s interviewees, data scientists might want to understand the inner-workings of his model, the importance of its features, as well as input-output dependencies. They might like to learn about robustness, data biases and how the model treats them, and performance issues to improve the model. On the other hand, managers might be more interested in being presented (by the data scientist) with a high-level analysis that they can understand on a business level but that nonetheless ensures them that they are not running a business or operational risk. While a regulator will focus on customer transparency and the avoidance of systemic risk, both of these aspects being highly model-agnostic.

These different requirements for different target audiences imply that a future tool for XAI needs to accomplish three things:

1) A rigorous, well-researched, and established approach to explainability that guarantees acceptance by all addressees.
2) Step-by-step reduction of the level of complexity, ranging from statistical, mathematical, and technical dependencies to relations understandable in terms of the business context (and that might even need to be model-agnostic).
3) A customisable (or pre-customized) visualization that conveys the exact “right amount of information” to the user facilitates an audience-dependent understanding almost intuitively.

Next to such a tool that unlocks the explainability of AI for different target audiences, more is needed to advance the wider adoption of AI in finance—in this, the participating interviewees of our study agreed. Regarding data quality and access to suitable talent, already a quite noticeable change is underway: As ever more data is available, institutions rapidly become more experienced and efficient in collecting, cleansing and processing of Big Data. At the same time, the “War for talents” is in full swing: Finance is competing with Big Tech for the next generation of data scientists and AI specialists. Thus driving demand which in turn has resulted in a growing number of graduates in these disciplines. Plus, financial institutions have already adapted to the new needs and demands of the young generations Y and Z. They are actively reshaping their employment policies, often mimicking Big Tech companies in this respect (McKinsey and Company Switzerland 2019).

On the other hand, the challenges regarding legacy IT systems and corporate culture are more difficult to overcome. Larger financial institutions and banks have started to digitize their business as early as the 1950 or 1960. Their processes are now being deeply intervened with and dependent on the IT infrastructure introduced 70 years ago. Thus, by today, every smaller amendment or even larger transition of the IT landscape of a bank comes along with a profusion of regulatory and operational risk restrictions. This dilemma is not an easy one to solve. Nevertheless, change is underway as it becomes increasingly apparent that there is no alternative to the adoption of AI in order to stay competitive. Finally, the necessary paradigm change in the corporate culture of financial institutions is a huge challenge, too. Finance is, and always has been, a rather traditional business in a highly regulated environment. Thus, mindsets prevalent in the financial industry are traditionally either diligent and conservative risk-manager-like profiles not prone to experiments of any sort or rather proactive managers motivated by business and profit considerations but with a limited understanding of and interest in complicated technical concepts. Furthering a culture of shared understanding between such profiles and the mindset of a gen-Y or gen-Z AI specialist is difficult. Yet, the shared motivation, the well-known incentives of the financial industry, the ever-ongoing generation change, and the competitive pressure exerted by FinTechs have started to transform how established financial institutions adopt new AI technologies.

Looking ahead, XAI faces even more challenges. As AI methodologies and implementations have become increasingly complex and involved, depending on ever-larger amounts of diverse data, the need for explainability in AI will become more pronounced and XAI itself more and more difficult. At the same time, this complexity and the “unexplainable part” of AI adds value beyond what humans or simple statistics can deliver. By leveraging and understanding the results AI presents us, we can gain new insights, understand the problem at hand in more detail, refine our analysis methods, and further accept this new technology concerning the whole spectrum of different target audiences.

## Data Availability

The datasets presented in this article are not readily available because The transcripts from the interviews are made available only to the researchers carrying out the study; Requests to access the datasets should be directed to hadj@zhaw.ch.
